# Social Structure Predicts Eye Contact Tolerance in Nonhuman Primates: Evidence from a Crowd-Sourcing Approach

**DOI:** 10.1038/s41598-020-63884-x

**Published:** 2020-04-24

**Authors:** Ethan G. Harrod, Christopher L. Coe, Paula M. Niedenthal

**Affiliations:** Department of Psychology, University of Wisconsin-Madison, Madison, Wisconsin USA

**Keywords:** Human behaviour, Animal behaviour

## Abstract

In most primates, eye contact is an implicit signal of threat, and often connotes social status and imminent physical aggression. However, in humans and some of the gregarious nonhuman primates, eye contact is tolerated more and may be used to communicate other emotional and mental states. What accounts for the variation in this critical social cue across primate species? We crowd-sourced primatologists and found a strong linear relationship between eye contact tolerance and primate social structure such that eye contact tolerance increased as social structures become more egalitarian. In addition to constituting the first generalizable demonstration of this relationship, our findings serve to inform the related question of why eye contact is deferentially avoided in some human cultures, while eye contact is both frequent and even encouraged in others.

## Introduction

Being looked at elicits a reflexive, involuntary response. Humans can detect that they are the target of another’s gaze through subcortical neural pathways, even without conscious awareness^1^. Detection of direct gaze in turn triggers a cascade of activation in social cognition centers of the brain that underlie the self-conscious state of arousal and endocrine responses we experience as the “feeling of being looked at”^2^. Achieving eye contact with another has important significance both for the sender of this social signal and the recipient. Among most primates, direct gaze can serve as an explicit and implicit signal of threat or dominance, indicating that overt physical aggression might soon follow^3–9^. It is thus often associated with social status and may elicit an avoidance response in a subordinate individual or an antagonistic counter response by another higher in the social hierarchy^3,5,10^. On the other hand, humans and members of some of the more gregarious primates, while still recognizing the context in which direct gaze signals threat, are more tolerant of direct gaze and respond by making eye contact^7,10–17^. In these species, eye contact may be used to communicate complex emotional and mental states, and to establish affiliative bonds^7,14,15,17,18^. What accounts for the fact that in some nonhuman primates direct gaze typically signals threat and does not invite eye contact, while in others eye contact plays a role in signaling nuanced features of a social interaction?

We conducted an investigation into the relationship between tolerance of eye contact and the nature of the social structure in which different primates live. In order to collect data that spanned numerous primate taxa, we used a novel crowd-sourcing method. Specifically, primatologists were surveyed about the social behavior of the animals that had been the subject of their scientific research for many years. Despite some limitations (discussed below), this method offers the benefits of control over definitions and uniform responses that represent extended observations over a career rather than during a single experiment or time period. We predicted that eye contact is tolerated more in primates that typically live in egalitarian groups with more social fluidity as compared to hierarchical groups with more rigid social stratification. Our *a priori* predictions were based on extant theory in social and cognitive psychology and derived from numerous previous ethological studies of nonhuman primates.

## Eye Contact

Eye contact is defined as the prolonged looking by one animal directly into another’s eyes^13^. It is distinct from gaze following, which also relies on the ability to detect and interpret gaze but with the purpose of mutually attending to an object during a cooperative task^10,19–21^. While eye contact and gaze following are undoubtedly related as components of a larger communicative repertoire, they serve different and distinct purposes among primates. Theories such as the cooperative eye hypothesis^22^ make the claim that gaze following (absent of head movement) has evolved as a distinctly human behavior with the purpose of supporting complex cooperative social tasks. Conversely, the use of eye contact as a signal of social intent is a behavior seen across the primate order. Due to these differences, we limit our conceptual grounding to research on eye contact as a social signal and recognize that the use of gaze following may not align completely with the use of eye contact in all primates.

## Eye Contact in Human Primates

Among humans, there are clear distinctions in how eye contact is used and tolerated, and these signaling and response patterns are socialized and entrained from early childhood. The initial basis for making eye contact starts soon after birth, with the ability to distinguish between direct and averted gaze arising in infants within 2–5 days after delivery. By 4 months of age, faces with a direct, as compared to averted, gaze elicit more attention and result in enhanced neural processing in infants^23^. Across cultures, eye contact between mothers and infants is initially actively sought and sustained^24,25^ and plays an important role in bonding with parents. However, as infants develop, caregivers socialize certain eye gaze patterns to encourage patterns of eye contact that are more congruent with culture-specific norms and expectations^24,26–28^.

The result of this shaping of looking behavior is that the meaning, use, and tolerance of eye contact in adults differs across cultures. For example, the frequency and duration of eye contact is lower in East Asian, than in Western European and American, cultures^13,29,30^. Japanese adults engage in significantly less eye contact than do either Canadians or Trinidadians during face-to-face interactions^31,32^. There is also cultural variation in how eye contact is interpreted, with East Asians, as compared to Western Europeans, viewing eye contact as more aggressive and signifying that a person is less approachable^29–31^. In addition, in many Asian as well as Middle Eastern cultures, the use of eye contact and staring is also a way to convey social status and rank, obliging subordinate individuals to avert or lower their gaze. Thus, there appear to be both universal and specific aspects to the meaning of direct gaze and eye contact which may correspond to how eye contact is used among nonhuman primates in more hierarchical versus egalitarian social systems.

Although the terms hierarchical and egalitarian as we understand them in the context of nonhuman primates are not commonly used to describe human social structures, we can still draw connections between nonhuman and human social structures by reference to the strategies used in accomplishing the social tasks common to group-living within these structures. Such tasks include negotiating one’s status in the social hierarchy, affiliative behaviors like consoling and trust building, and responding to unfamiliar and unrelated conspecifics. Human cultures and societies with tighter social norms and more rigid status social hierarchies, such as Japan, China, India, and Malaysia, employ strategies more comparable to those used in the hierarchical social structures seen in some nonhuman primates^33^. Conversely, cultures in which norms are looser and less based in explicit assertion and maintenance of social status, can be likened to the social structures of the more egalitarian primates, such as the bonobo communities and muriqui troops.

## Eye Contact in Nonhuman Primates

The idea that eye contact conveys primarily an agonistic message in nonhuman primates has been supported by many experimental studies in rhesus macaques using photographs of direct gazing monkeys or human faces with wide-eyed, intentional looking as a threatening stimulus^20,34,35^. However, the conclusions from this research generalize to additional primate taxa, including many prosimians, most monkeys, and the great apes, all of which use a directed stare as a threatening signal^10,36,37^. Especially among those primates that live in groups with clearer dominance hierarchies, the use of direct gaze is a means to convey higher social status and to exert control over the behavior and proximity of subordinates. In contrast, eye contact is used infrequently and typically not prolonged during joint action in many of these primates^10,15,16,19,38^. As an example, rhesus macaques typically make eye contact in order to impose their social standing or to challenge a conspecific, most often leading to avoidance and spatial displacement by the subordinate^6,18,38^. Even when engaging in prosocial behaviors such as reconciliation and conciliatory behavior, rhesus macaques often avoid eye contact^17,18^. Similar findings have been obtained in the mostly solitary and territorial mouse lemurs, with prolonged eye contact initiating aggressive encounters, but avoided during affiliative behaviors or when first encountering an unfamiliar conspecific^36^.

In contrast, the more gregarious bonobo rarely engages in affiliative behaviors without first establishing eye contact^16,39^. In bonobos, marmosets that live in small family groups, and some species of macaques, such as the stumptailed and tonkean macaques, eye contact is made more regularly in order to initiate non-agonistic social interactions, establish and maintain affiliative bonds, to initiate play, and serves to aid in cohesion when animals are searching for food and resources, even while a more prolonged direct stare is maintained in the behavioral repertoire as a threat signal^16,17,40–42^.

Among nonhuman primates, the distinction between the use of eye contact as a threat or an affiliative signal appears to follow a logical pattern that is congruent with both their phylogenetic relationships and the nomenclature used to categorize different social group structures^10^. Thus, rather than just focusing on gaze behavior in one type of primate and ascribing it to inherent dispositions of functions within that particular species^18^, we sought to generate a larger, overarching and parsimonious framework that could better inform research on this communicative behavior among humans.

## Results

**Figure 1:**
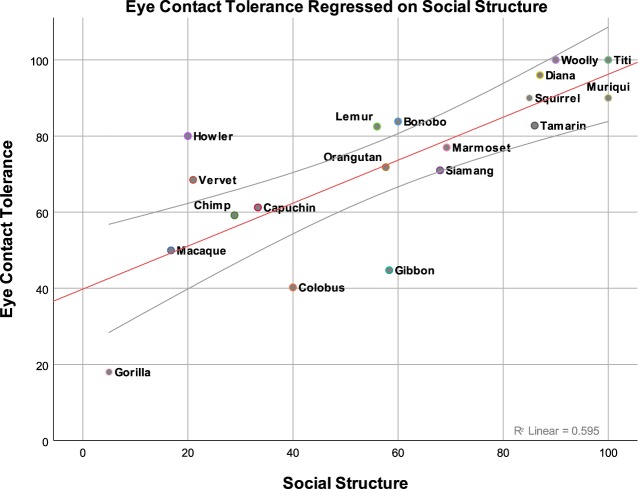
Significant positive relationship between expert ratings of primate-typical tolerance for eye contact and the more stratified or affiliative attributes of their social relationships (hierarchical versus more egalitarian). On the x-axis, a 0 (completely hierarchical) to 100 (completely egalitarian) scale of primate social structure. On the y-axis, a 0 (no tolerance) to 100 (high tolerance) scale of primate eye contact tolerance. This figure is generated from a data set consisting of 55 data points. For the sake of interpretability, these 55 data points have been collapsed into the shown 19 data points. Each point represents a specific grouping of primates as indicated by their labeling.

As predicted, there was a strong positive correlation between the rating of eye contact tolerance and the typical group social structure exhibited by the 19 primate taxa sampled, *r* = 0.528, *t*(52) = 4.49, *p* < 0.001. To further assess the relationship between eye contact tolerance and social structure, we estimated a linear mixed effect model in which eye contact tolerance was regressed on social structure (cluster means) as well as social structure (centered within clusters). A by-primate random intercept and a by-primate random slope for social structure (centered within clusters) was included in the model. As predicted, there was a significant relationship between social structure (cluster means) and eye contact tolerance, *b* = 0.538, *F*(1, 13.8) = 14.6, *p* = 0.002 (Fig. 1), such that eye contact was tolerated to a greater degree among primates that have more egalitarian, as compared to hierarchical, social structures.

To ensure that this effect was not driven by differences in the environment in which the primates were observed, a linear mixed effects model was estimated in which eye contact tolerance was regressed on social structure (cluster means), social structure (centered within clusters), observational setting (captive versus field), and the interaction between social structure (cluster means) and observation context. A by-primate random intercept and a by-primate random slope for both social structure (centered within clusters) and observation context were included in the model. Notably, there was no main effect of observational setting on reported eye contact tolerance, *p* = 0.802, and the effect of social structure (cluster means) on eye contact was not moderated by the context in which the primates were observed, *p* = 0.812.

**Figure 2:**
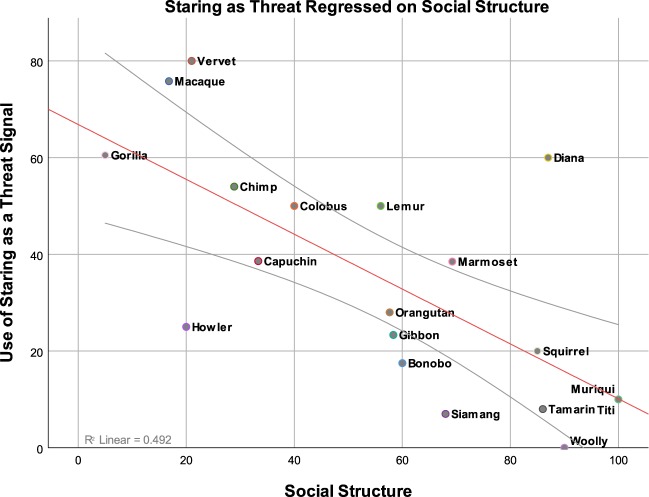
Significant positive relationship between expert ratings of primate-typical use of staring as a threat signal and the more stratified or affiliative attributes of their social relationships (hierarchical versus more egalitarian). On the x-axis, a 0 (completely hierarchical) to 100 (completely egalitarian) scale of primate social structure. On the y-axis, a 0 (no use) to 100 (exclusive use) scale of primate use of staring as a threat signal. This figure is generated from a data set consisting of 55 data points. For the sake of interpretability, these 55 data points have been collapsed into the shown 19 data points. Each point represents a specific grouping of primates as indicated by their labeling.

**Figure 3:**
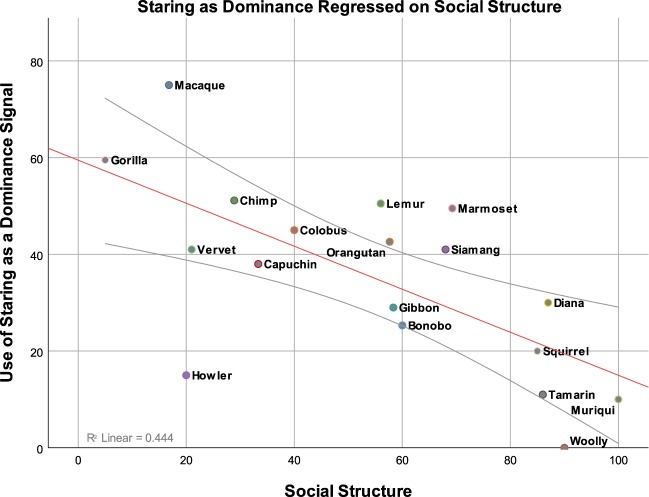
Significant positive relationship between expert ratings of primate-typical use of staring as a dominance signal and the more stratified or affiliative attributes of their social relationships (hierarchical versus more egalitarian). On the x-axis, a 0 (completely hierarchical) to 100 (completely egalitarian) scale of primate social structure. On the y-axis, a 0 (no use) to 100 (exclusive use) scale of primate use of staring as a dominance signal. This figure is generated from a data set consisting of 55 data points. For the sake of interpretability, these 55 data points have been collapsed into the shown 19 data points. Each point represents a specific grouping of primates as indicated by their labeling.

The potential relationship between social structure and the use of staring as a threat or dominance signal was also tested in two separate linear mixed effect models in which threat and dominance were regressed on social structure (cluster means) and social structure (centered within clusters). A by-primate random intercept and a by-primate random slope for social structure (centered within clusters) was included. Importantly, there was a significant relationship between social structure (cluster means) and the use of staring as a threat signal, *b* = −0.663, *F*(1, 14.8) = 15.4, *p* = 0.001 (Fig. 2.), as well as the use of staring behavior as a dominance signal, *b* = −0.503, *F*(1, 15.3) = 7.99, *p* = 0.012 (Fig. 3.). The frequency with which staring is used as a signal of threat or dominance was far lower among primates with more egalitarian, as compared to hierarchical, social structures.

Additionally, due to the novelty of the crowd-sourcing method used, the reliability of the surveyed expert ratings posed a potential limitation and concern for the validity and reliability of the above findings. To bolster confidence in the reliability of the expert ratings and thus the findings, the inter-rater reliability of the expert responses was estimated with multiple intraclass correlations (ICC) generated for each primate rated by more than one expert. ICC estimates and their 95% confidence intervals were calculated using the “icc” package in RStudio based on a mean-rating, absolute-agreement, 1-way random-effects model (Table 1). An average ICC of 0.755, *t* = 8.22, *p* < 0.001, LoCI = 0.627, UpCI = 0.868, was calculated from the coefficients to provide an estimate of the average reliability of the expert responses. We also calculated an average ICC of 0.913, *t* = 11, *p* < 0.001, LoCI = 0.816, UpCI = 0.959, from only the primates with significant ICCs. The t values and 95% confidence intervals for these coefficients were calculated through a Fisher Transformation. Based on these ICCs, we have compelling evidence to conclude there was moderate to good^43^ within-taxa reliability in the expert ratings of eye contact tolerance and social structure.

**Table 1:** Descriptive statistics for the intraclass correlations for each primate rated by more than one expert. Confidence intervals for the average intraclass correlation coefficients were calculated through a Fisher Transformation.

Primates	*n*	Intraclass Correlations			
		*ICC*	*p* =	*LoCI*	*UpCI*
*Howler*	2	0.905	0.023*	0.047	0.994
*Capuchin*	6	0.785	0.056*	−0.49	1
*Marmoset*	4	0.594	0.112	−0.815	0.972
*Vervet*	2	0.863	0.042*	−0.363	0.991
*Lemur*	2	0.582	0.632	−14.78	0.895
*Gorilla*	2	0.992	<0.001***	0.919	0.999
*Gibbon*	3	0.531	0.200	−2.407	0.988
*Rhesus Macaque*	10	0.937	<0.001***	0.78	0.996
*Chimpanzee*	8	0.688	0.090	−0.96	1
*Bonobo*	3	0.93	0.001**	0.62	0.995
*Orangutan*	3	0.517	0.183	−1.62	0.967
*Tamarin*	2	0.982	0.001**	0.818	0.999
*Average ICC*	47	0.775	<0.001***	0.627	0.868
*Average ICC of Sig. ICCs*	27	0.913	<0.001***	0.816	0.959

## Discussion

The present findings document, for the first time, a positive linear relationship between the tolerance of eye contact and the extent to which the social relationships of primates are more egalitarian across a diverse and representative sampling of nonhuman primates. A novel crowd-sourcing method of data collection was employed, which proved to be reliable and highly efficient in that each data point encompassed many years of observations for each primate. In total, the survey method provided information about the social and communicative behavior of 19 different primate taxa.

Despite its novel methodology and clear findings, the study is not without limitations. First, while the data were acquired from a large number of experts (*n* = 55), the number of reports for each primate was not evenly distributed (see Tables 2–3 for the exact number of reports per primate). For six of the 19 primates, ratings were obtained from only one expert, lessening the reliability of these singular responses. However, it should be reiterated that the conclusions drawn from our findings are based on cross-taxa comparisons, not individual scores. In addition, the intraclass correlations described above revealed largely positive correlations indicative of the reliability of the survey methodology and expert ratings.

**Table 2:** Rating of the hierarchical nature of social interactions and the tolerance of eye contact for each primate. Social structure is scored on a 0 (completely hierarchical) to 100 (completely egalitarian) point scale. Eye contact tolerance is scored on a 0 (no tolerance) to 100 (high tolerance) point scale.

Primates	*n*	Social Structure Rating		Eye Contact Tolerance	
		*M*	*SD*	*M*	*SD*
*Howler*	2	20	14.14	80	0
*Muriqui*	1	100	NA	90	NA
*Titi*	1	100	NA	100	NA
*Capuchin*	6	33.33	22.29	61.25	22.52
*Marmoset*	4	69.25	40.57	77	13.26
*Diana*	1	87	NA	96	NA
*Vervet*	2	21	1.41	68.5	2.12
*Colobus*	2	40	14.14	40.25	14.5
*Lemur*	2	56	36.77	82.5	24.75
*Gorilla*	2	5	7.07	18	1.41
*Gibbon*	3	58.33	28.43	44.75	3.89
*Woolly*	1	90	NA	100	NA
*Rhesus Macaque*	10	16.8	14.79	49.95	30.31
*Chimpanzee*	8	28.88	36.71	59.19	29.71
*Bonobo*	3	60	17.32	83.83	13.29
*Orangutan*	3	57.67	6.81	71.83	20.25
*Tamarin*	2	86	5.66	82.75	10.25
*Siamang*	1	68	NA	71	NA
*Squirrel*	1	85	NA	90	NA

**Table 3:** Rating of the use staring as a signal of threat and dominance for each primate. Use of staring is scored on a 0 (never used) to 100 (exclusively used) point scale.

Primates	*n*	Use of Staring to Signal Threat		Use of Staring to Signal Dominance	
		*M*	*SD*	*M*	*SD*
*Howler*	2	25	21.21	15	7.07
*Muriqui*	1	10	NA	10	NA
*Titi*	1	10	NA	0	NA
*Capuchin*	6	38.6	39.11	38.39	39.62
*Marmoset*	4	38.5	38.73	49.5	43.87
*Diana*	1	60	NA	30	NA
*Vervet*	2	80	7.07	41	26.87
*Colobus*	2	50	28.28	45	7.07
*Lemur*	2	50	14.14	50.5	28.99
*Gorilla*	2	60.5	0.71	59.5	0.71
*Gibbon*	3	23.33	14.64	29	21.63
*Woolly*	1	0	NA	0	NA
*Rhesus Macaque*	10	75.8	16.82	75	16.59
*Chimpanzee*	8	54	35.69	51.14	30.89
*Bonobo*	3	17.5	3.54	25.33	18.58
*Orangutan*	3	28	23.52	42.67	32.62
*Tamarin*	2	8	2.83	11	11.31
*Siamang*	1	7	NA	41	NA
*Squirrel*	1	20	NA	20	NA

A second consideration is that while responses were solicited from professional scientists for individual primates, we cannot claim that the area of expertise of each scientist was eye contact or social structure. We polled primatologists without regard for expertise in these topics because social signaling and structure relate to most every primate behavior in some manner. Despite our attempt to sample without bias, it is still possible that some ratings were based on commonly held notions, even stereotypes, about the species. Notably, however, for species that were rated by two or more scientists, there was reassuring concordance in responses (see Table 1). Future studies can use more precise terminology or provide an exemplar, such as the commonly studied rhesus macaque as a reference point. Such an approach will further reduce the potential for bias in expert reports.

We also recognize that questions remain about the validity of the present definitions of social structure and eye contact tolerance. Although we are not aware of a comprehensive dataset of eye contact tolerance across primates that would allow us to directly compare our findings, there is an extant literature on the sociality and communicative styles of the 19 sampled primates. For example, a comparison of pre-existing reports on the sociality (hierarchical vs. egalitarian) of multiple species of macaques demonstrated significant species differences in counter-aggression and hierarchical steepness of their social groups^20^. Importantly, those differences are consistent with our findings, such that more hierarchical, as compared to egalitarian, macaque species engaged in the lowest rates of counter-aggression and reconciliatory behavior. One study noted difference in the use of facial displays between the tufted and the white-faced capuchin that were consistent with their differences in sociality^44^. While another study examining the sociality of white-faced capuchins, ranked them as intermediate in social style (partly hierarchical and partly egalitarian), and noted moderate tendencies toward aggressive, reconciliatory, and kin-biased behavior^45^. These findings also are in keeping with our experts’ reports, but now within a particular species. Within apes, it has been shown that bonobos follow the gaze of other primates most frequently, followed by chimpanzees and then gorillas^22^. Notably, the order of this ranking is consistent with our ratings of eye contact tolerance for the same primates. Finally, the differences in sociality and reconciliatory behavior in the two species within the genus Pan, chimpanzees and bonobos^16,39^, concur with the predictions from our model generated across the Primate Order. Taken together, we feel these findings provide compelling support for the validity of the expert ratings for primate social structure and eye contact tolerance.

Further, although the survey questions requested that the professional primatologist list a species name, most experts provided the common name or the genus. Therefore, for this analysis, the genus and common names are provided. While grouping primates in this manner resulted in a model generated at a phylogenetic level, and we appreciate that there exists species variation within a genus, we believe that it may have helped to extract the general principles across the primate order. As a result, this report provides a clear estimate of how the relationship between eye contact and sociality typically occurs across the Primate Order, enabling future research to elucidate differences at the species level. Such research will both help to identify those primates that adhere to the general pattern identified in this report, as well as to determine the reasons for those species that deviate either in their social behavior or use of staring and eye contact as signals.

Finally, although our analyses focused on eye contact behavior in general, visual signaling among primates is most certainly influenced by context. An individual may be more tolerant of eye contact in one situation with a familiar and related conspecific but have little to no tolerance of looking behavior by an unfamiliar animal. Despite distinguishing between eye contact behavior and staring, as well as differences in eye contact behavior during approach and when approached, this nuance was not fully captured by our survey. We recognize that the survey did not address the flexible use of eye contact, or even purposeful deviation from species-typical behavior. However, the primary aim was to extract general principles on a broader level, which would lay the foundation for others to interrogate these questions more specifically in controlled studies. Future research will examine such questions.

The present findings provide the basis for hypothesis-generation about the reasons for variation in eye contact use among humans, including the negotiation and maintenance of social status in more hierarchical social systems. Primates, such as the muriqui and bonobo, who live in relatively fluid, egalitarian social groups are faced with the alternative scenario of needing to engender social cohesion and cooperation^16,42^. Similar variation in the social norms and expectancies occur across human cultures. One solution to these social challenges would be to allow internal states and intentions to be known through eye contact and other non-verbal gestures, behavior that is used universally by all humans because of our common ancestry, but in culture specific ways^46^. The current survey has advanced our understanding of the basis for this link between communicative behavior and sociality and informs about the ancestral antecedents of eye contact behavior among humans that can be used to examine its use in different cultures and countries.

## Method

Fifty-five experts in primatology were recruited through the International Primatological Society (IPS), as well as email solicitation, on the basis of their reputation as experts for particular species, with the goal to represent all of the major taxa in the Primate Order. The survey included scientists who studied animals under natural conditions as well as in captive settings. They were queried with a standardized set of questions that asked about both typical eye contact behavior and the sociality of the species. In order to be considered an expert, respondents were required to be researchers in the field of primatology, possessing a master’s degree or Ph.D, and affiliated with an academic institution or accredited zoo. Fifty-three of the fifty-five respondents were affiliated with an academic institution. The remaining two respondents were affiliated with an accredited zoo. Responses from individuals that did not meet these requirements, or whose identity could not be verified, were not included in our analyses. Primate social structure was scored on a scale from 0 to 100, where 0 indicated a completely hierarchical social system and 100 indicated a completely fluid, egalitarian social system, such as the fission/fusion sociality of the spider monkey or muriqui. The central outcome variable, eye contact tolerance, was polled on two different scales, from 0 (no tolerance) to 100 (high tolerance). The first inquired about eye contact tolerance when approaching a conspecific, and the other inquired about eye contact tolerance when being approached by a conspecific. We observed a strong positive correlation between the two ratings of eye contact tolerance, *r* = 0.909, *t*(51) = 15.6, *p* < 0.001. Based on the high similarity in the ratings, we averaged the two sets of scores into a composite variable labelled Eye Contact Tolerance. This composite score served as the main outcome variable in our analyses. The degree to which staring is used a threatening signal and a signal of dominance was polled through two separate questions on a scale from 0 (never) to 100 (exclusively). Respondents also indicated whether their judgments were based on laboratory or field observations. In total, reports were received on the behavior of 19 different primates.

The present study’s methodology and experimental procedure were reviewed and approved by the University of Wisconsin Madison institutional review board (IRB), ID 2018–0736. This approval extends to the recruitment, email solicitation, and use of the surveyed information, all of which was performed in accordance with the guidelines and regulations set by the UW-Madison IRB. Participants were informed about the general goals of the study and provided their informed consent for use of the acquired data. The present study was given IRB exemption from the requirements to acquire signed consent and to provide debriefing on the full extent of the study goals.

## Data Availability

All data and code used in the analyses and figure creation has been made publicly available on the Open Science Framework (OSF) website. Data can be accessed with the following 10.17605/OSF.IO/YBHAQ. Accessing this page will grant access to both the collapsed dataset (scores averaged across primates) and the full dataset (containing individual scores). Both data sets were used in the analyses. Figure one was created with the full dataset, but data points from the collapsed dataset are plotted for viewing clarity. Statistical tests (as detailed in the results section) were completed using the full data set. Please contact us at eharrod@wisc.edu with any questions regarding the data or analysis code.

## References

[1] Stein T, Senju A, Peelen MV, Sterzer P (2011). Eye contact facilitates awareness of faces during interocular suppression. Cognition..

[2] Senju A, Johnson MH (2009). The eye contact effect: mechanisms and development. Trends in Cognitive Sciences..

[3] Van Hoof J. A. R. A. M. The facial displays of the Catarrhine monkey and apes. In Primate ethology. Chicago: Aldine, (1967).

[4] Lorenz K., *On aggression*. New York: Harcourt, Brace & World, (1966).

[5] Ellsworth P, Carlsmith JM (1973). Eye contact and gaze aversion in an aggressive encounter. Journal of Personality and Social Psychology..

[6] Perrett D. I. & Mistlin A. J. Perception of facial characteristics by monkeys. In *Comparative Perception*, New York: John Wiley, p. 187±215 (1991).

[7] Emery NJ (2000). The eyes have it: the neuroethology, function and evolution of social gaze. Neuroscience & Biobehavioral Reviews..

[8] Andrew R. J., The displays in the Primates. In *Evolutionary and Genetic Biology of the Primates*, vol. 2. (New York: Academic Press, 1964).

[9] Kobayashi H, Kohshima S (2001). Unique morphology of the human eye and its adaptive meaning: comparative studies on external morphology of the primate eye. Journal of Human Evolution..

[10] Gomez J. C. Ostensive behavior in great apes: the role of eye contact. In *Reaching Into Thought: The Minds of the Great Apes* (Cambridge University Press), pp. 131-151 (1998).

[11] Argyle M. & Cook M. *Gaze and mutual gaze* (Cambridge U Press, Oxford, England, 1976), Gaze and mutual gaze.

[12] Argyle M, Dean J (1965). Eye-contact, distance and affiliation. Sociometry..

[13] Kleinke CL (1986). Gaze and eye contact: A research review. Psychological Bulletin..

[14] Kobayashi H, Kohshima S (2001). Unique morphology of the human eye and its adaptive meaning: comparative studies on external morphology of the primate eye. Journal of Human Evolution..

[15] Yamagiwa J (1992). Functional analysis of social staring behavior in an all-male group of mountain gorillas. Primates..

[16] Kano K, Hirata S, Call J (2015). Social Attention in the two species of pan: Bonobos make more eye contact than chimpanzees. PLOS ONE..

[17] De Waal F. B. *Peacemaking among primates*. Harvard University Press (1989).

[18] De Waal FB, Yoshihara D (1983). Reconciliation and redirected affection in rhesus monkeys. Behaviour..

[19] Kano K, Call J (2014). Cross-species variation in gaze following and conspecific preference among great apes, human infants and adults. Animal Behaviour..

[20] Tomasello M, Call J, Hare B (1998). Five primate species follow the visual gaze of conspecifics. Animal Behaviour..

[21] Vick S-J, Bovet D, Anderson J (2001). Gaze discrimination learning in olive baboons (Papio anubis). Animal Cognition..

[22] Tomasello M, Hare B, Lehmann H, Call J (2007). Reliance on head versus eyes in the gaze following of great apes and human infants: the cooperative eye hypothesis. Journal of human evolution.

[23] Farroni T, Csibra G, Simion F, Johnson MH (2002). Eye contact detection in humans from birth. Proceedings of the National Academy of Sciences..

[24] Bullowa M. *Before Speech: The Beginning of Interpersonal Communication* (CUP Archive, 1979).

[25] Keller H., Poortinga Y. H., Butterworth G., Scholmerich A. & Schölmerich A. *Between Culture and Biology: Perspectives on Ontogenetic Development* (Cambridge University Press, 2002).

[26] Keller H, Scholmerich A, Eibl-Eibesfeldt I (1988). Communication patterns in adult-infant interactions in western and non-western cultures. Journal of Cross-Cultural Psychology..

[27] Papoušek H. & Papoušek M. Intuitive parenting: A dialectic counterpart to the infant’s integrative competence in *Handbook of infant development, 2nd ed* (John Wiley & Sons, Oxford, England), *Wiley series on personality processes*, pp. 669–720 (1987).

[28] Trevarthen C, Aitken K (2001). J., Infant Intersubjectivity: Research, theory, and clinical applications. Journal of Child Psychology and Psychiatry..

[29] Akechi H (2013). Attention to eye contact in the west and east: Autonomic responses and evaluative ratings. PLOS ONE..

[30] Blais C, Jack RE, Scheepers C, Fiset D, Caldara R (2008). Culture shapes how we look at faces. PLOS ONE..

[31] McCarthy A, Lee K, Itakura S, Muir DW (2006). Cultural display rules drive eye gaze during thinking. Journal of Cross-Cultural Psychology..

[32] McCarthy A, Lee K, Itakura S, Muir DW (2008). Gaze display when thinking depends on culture and context. Journal of Cross-Cultural Psychology..

[33] Gelfand MJ (2011). Differences between tight and loose cultures: A 33-nation study. science.

[34] Teufel C, Gutmann A, Pirow R, Fischer J (2010). Facial expressions modulate the ontogenetic trajectory of gaze-following among monkeys. Developmental Science.

[35] Gothard KM, Erickson CA, Amaral DG (2003). How do rhesus monkeys (Macaca mulatta) scan faces in a visual paired comparison task?. Animal Cognition.

[36] Coss RG (1978). Perceptual determinants of gaze aversion by the lesser mouse lemur (Microcebus murinus), the role of two facing eyes. Behaviour..

[37] Sapolsky RM (2005). The influence of social hierarchy on primate health. Science..

[38] Sato, N. & Nakamura, K. Detection of directed gaze in rhesus monkeys (Macaca mulatta). *Journal of Comparative Psychology*, **115**, 115 (2001). 10.1037/0735-7036.115.2.115 11459157

[39] Savage, E. S. & Bakeman R., Sexual morphology and behavior in Pan paniscus. In: Recent Advances in Primatology, Academic Press, New York, pp. 613 – 616 (1978).

[40] Matsumura S (1999). The evolution of “egalitarian” and “despotic” social systems among macaques. Primates..

[41] Mitchell JF, Leopold DA (2015). The marmoset monkey as a model for visual neuroscience. Neuroscience Research..

[42] Russon A. E., Bard K. A. & Parker S. T. *Reaching Into Thought: The Minds of the Great Apes* (Cambridge University Press, 1998).

[43] Koo TK, Li MY (2016). A guideline of selecting and reporting intraclass correlation coefficients for reliability research. Journal of chiropractic medicine.

[44] De Marco A, Petit O, Visalberghi E (2008). The repertoire and social function of facial displays in cebus capucinus. International Journal of Primatology.

[45] Bergstrom ML, Fedigan LM (2013). Dominance style of female white‐faced capuchins. American journal of physical anthropology.

[46] Niedenthal PM, Rychlowska M, Zhao F, Wood A (2019). Historical migration patterns shape contemporary cultures of emotion. Perspectives on Psychological Science.

